# Atypical Biopsy-Proven Myoglobin-Induced Acute Tubular Injury in the Setting of Normal Creatine Phosphokinase and Urine Myoglobin

**DOI:** 10.7759/cureus.103462

**Published:** 2026-02-12

**Authors:** Amit Ramrattan, Barry M Wall, Hieu Q Vo, Adedamola Adeboye

**Affiliations:** 1 Department of Internal Medicine, Port of Spain General Hospital, Port of Spain, TTO; 2 Department of Nephrology, Veterans Affairs Medical Center, Memphis, USA; 3 Department of Medicine, Division of Med Nephrology, The University of Tennessee Health Science Center, Memphis, USA

**Keywords:** acute kidney failure, creatine phosphokinase (cpk), influenza vaccine, myoglobin cast nephropathy, rhabdomyolysis

## Abstract

Rhabdomyolysis can be complicated by acute kidney injury due to myoglobin-induced nephrotoxicity and tubular injury, in addition to volume depletion related to fluid sequestration. It is, however, unusual to have acute kidney injury with normal creatine phosphokinase (CPK) and negative urine myoglobin. We present an atypical case in which a 66-year-old woman presented with acute renal failure of unclear etiology, which required temporary dialysis support. After extensive evaluation, a renal biopsy was conclusive for myoglobin-induced tubular injury. This patient had received an influenza vaccine one month prior to her presentation, which was complicated by severe myalgias, suggesting that she had significant rhabdomyolysis in the initial days following the vaccination, which led to the acute kidney injury. CPK was normal and urine myoglobin was negative at the time of presentation, one month following vaccination. This is an uncommon presentation of rhabdomyolysis and acute kidney injury and demonstrates that acute kidney injury due to rhabdomyolysis can have a delayed presentation, which can be confirmed by kidney biopsy findings of acute tubular injury with myoglobin staining casts.

## Introduction

Rhabdomyolysis is a syndrome that is characterized by the breakdown of skeletal muscle with the subsequent release of creatine phosphokinase (CPK), myoglobin, lactate dehydrogenase, protein, and electrolytes into the circulation [1]. CPK rises 2-12 hours after the initial muscle injury, peaks in 1-3 days, and then declines over 3-5 days. Myoglobin, in contrast, increases rapidly but is cleared quickly through renal excretion with a normal level re-established within 24 hours [2]. Early testing, therefore, can diagnose rhabdomyolysis, but delayed presentations can make the diagnosis unclear. Acute kidney injury (AKI) occurs due to the deleterious effects of myoglobin, which exerts its damaging effects via tubular obstruction, renal vasoconstriction, and tubular damage by oxidative injury [3]. AKI secondary to rhabdomyolysis was first described by Bywaters and Beall in 1942 [4], and it is estimated that 10%-40% of patients with rhabdomyolysis develop AKI and up to 15% of all cases of AKI can be attributed to rhabdomyolysis [5]. This syndrome can be seen because of trauma, drugs, toxins, infections, muscle ischemia, metabolic and genetic disorders, exertion or prolonged bed rest, temperature-induced states, and malignant hyperthermia [6]. Although the influenza vaccine is known to cause local and systemic effects such as pain, fever, headache, fatigue, and myalgia, rhabdomyolysis is a rarely documented side effect [7,8]. We present a very unusual case of an AKI that was a result of a very late presentation of rhabdomyolysis that was possibly triggered by an influenza vaccine. This delayed presentation meant that the CPK levels were normal, and a kidney biopsy with myoglobin immunostaining proved essential in the diagnosis.

## Case presentation

The patient is a 66-year-old African American woman who has a past medical history of morbid obesity, type 2 diabetes mellitus complicated with peripheral neuropathy, hypertension, hyperlipidemia, microcytic anemia, and asthma in addition to a past surgical history of a cholecystectomy six years ago. Her home medications include the following: aspirin 81 mg daily, rosuvastatin 20 mg daily, lisinopril 5 mg daily, amlodipine 10 mg daily, metformin 850 mg daily, glipizide 10 mg daily, gabapentin 300 mg daily, fluticasone/salmeterol 250/50 mcg inhaler, and ferrous sulfate 325 mg daily. Her as-needed medications include loratadine 10 mg, famotidine 40 mg, and Lasix 20 mg for leg swelling. One month prior to her presentation to the hospital, she had seen her primary care physician (PCP), had a complete blood count (CBC) and urinalysis tests done, and received Seqirus Flucelvax 0.5 mL on the left deltoid. On that day, she reported no weakness or myalgia to her PCP. Table 1 shows her lab investigations from her PCP visits prior to and on presentation to the hospital.

**Table 1:** Laboratory investigations and results. MCV: mean corpuscular volume

Test description	Reference range	7 months prior to presentation	1 month prior to presentation	Admission investigations
White blood cell	3.4-10.8 x 10^3^/µL	5.7	6.9	11.2
Hemoglobin	11.1-15.9 g/dL	9.8	8.0	7.0
MCV	79-97 fL	68	65	61.4
Hematocrit	34.0%-46.6%	34.5	30.2	25.9
Platelets	150-450 x 10^3^/µL	459	499	409
Reticulocyte %	0.5%-2.0%	-	-	0.44
Absolute reticulocytes	0.03-0.09 x 10^6^/µL	-	-	0.016
Sodium	136-145 mmol/L	142	-	137
Potassium	3.5-5.1 mmol/L	3.5	-	2.8
Total carbon dioxide	22-29 mmol/L	-	-	23
Anion gap	5-20	-	-	17
Blood urea nitrogen	9.8-20.1 mg/dL	5	-	40.0
Creatinine	0.56-1.11 mg/dL	0.67	-	9.13
Calcium	8.4-10.2 mg/dL	9.1	-	9.1
Albumin	3.5-5.0 g/dL	4.1	-	3.0
Total protein	6.4-8.3 mg/dL	6.7	-	6.7
Creatine kinase	29-168 unit/L	-	-	133
Uric acid	2.6-6.0 mg/dL	-	-	9.0
Procalcitonin	<0.50 ng/mL	-	-	0.84
Iron	50-170 mcg/dL	-	-	18
Transferrin	173-360 mg/dL	-	-	248
Ferritin	4.6-204 ng/mL	-	-	10
A1C	<5.5%	7.6	-	8.4
Parathyroid hormone	14.5-87.1 pg/mL	-	-	178
Thyroid-stimulating hormone	0.35-4.94 µIU/mL	1.85	-	0.29
Anti-nuclear antibody	Negative	-	-	Negative
Double-stranded DNA antibody	≤4 IU/mL	-	-	1
C3 complement	83.0-193.0 mg/dL	-	-	147
C4 complement	15.0-57.0 mg/dL	-	-	40.6
Kappa free light chain	3.3-19.4 mg/L	-	-	131.3
Lambda free light chain	5.7-26.3 mg/L	-	-	61.2
Kappa:lambda ratio	0.26-1.65	-	-	2.15
Myeloperoxidase antibody	<1.0 index value	-	-	<0.2
Proteinase antibody	<1.0 index value	-	-	<0.2
Glomerular basement membrane antibody	0.0-0.9 units	-	-	<0.2
Urine albumin/creatinine	<30 mg/g	34	-	-
Urinalysis/microscopic examination				
Appearance	Clear	Clear	Cloudy	Turbid
Protein	Negative/trace	1+	2+	3+
Glucose	Negative	Negative	Negative	1+
Ketone	Negative	Negative	Negative	Negative
Blood	Negative	Negative	Negative	2+
Nitrite	Negative	Negative	Negative	Negative
Red blood cells	0-2/hpf	None seen	0-2	182
White blood cells	0-5/hpf	0-5	0-5	>182
Cast/type	None seen	None seen	Present/hyaline	Hyaline cast
Bacteria	None/few seen	None seen	None seen	1+
Urine myoglobin	0-13 ng/mL	-	-	2
Protein/creatinine ratio	<0.15 mg/g	-	-	7,087

The investigations prior to hospitalization were in keeping with diabetic kidney disease, as shown by the presence of microalbuminuria. She had a normal lipid panel and liver function tests seven months prior to the hospital presentation but had no prior CPK done.

She presented to the Emergency Department following a mechanical fall after experiencing what she described as weakness in her knees. On further questioning, she mentioned that she developed generalized myalgia that began two to three days after receiving her flu vaccine. Myalgia began to improve after two weeks, and then for the following two weeks, she experienced weakness, described "beer" colored, foamy urine without an abnormal smell, and endorsed decreased output and urgency without dysuria. She denied using non-steroidal anti-inflammatory drugs (NSAIDs) and illicit drugs such as cocaine and reported compliance with her regular medications, which included rosuvastatin. Admission vitals showed a blood pressure of 110/57 mmHg, pulse rate of 97 beats per minute, temperature of 37.1°C (98.8°F), respiratory rate of 18 breaths per minute, height of 1.6 m (5'3"), weight of 99.8 kg (220 lbs), and SpO_2_ of 100%. Her physical examination was normal except for mucosal pallor, and there was no muscle tenderness or swelling. Her CT head showed no acute pathology. She had a bladder scan, which was negative for urinary retention.

It is important to highlight that there was no biochemical evidence of rhabdomyolysis, given normal creatine kinase and negative urine myoglobin. Her anemia was likely due to iron deficiency, as her ferritin level was low in addition to her reticulocyte count. There was no evidence of shock as shown by her admission vitals, and her borderline elevated procalcitonin made sepsis or bacterial infection etiology unlikely. At this point, the subjective decreased urine output with no evidence of urinary retention, severe renal impairment, and her urinalysis with red blood cells, white blood cells, and nephrotic range proteinuria pointed to a possible glomerular pattern of AKI. One differential could be severe diabetic nephropathy, though one would not expect red or white blood cells to be present. Another differential could be acute interstitial nephritis from her famotidine, but this was unlikely as her CBC was negative for peripheral eosinophilia. The presence of both red and white blood cells can also suggest a urinary tract infection, but a urine culture was negative for bacterial growth. The presence of sterile pyuria in this case made a systemic inflammatory condition with renal involvement a top differential diagnosis at the time.

Renal ultrasound was normal, the right kidney was 13.2 cm in length with cortical thickness of 1.7 cm, and the left kidney was 12.9 cm in length with cortical thickness of 1.9 cm. A renal biopsy was done, after which a temporary vascular catheter was placed to initiate dialysis, as she remained oliguric by daily urine measurements < 300 mL and was developing peripheral edema. After the renal biopsy was done, she was given three days of pulsed methylprednisolone 1 g daily for three days. The reason for this empirical treatment was due to a systemic inflammatory condition being the main differential diagnosis at the time, as her anti-neutrophil cytoplasm antibody (ANCA), anti-glomerular basement membrane (anti-GBM) results, and kidney biopsy results were eagerly awaited. Her hemoglobin had also decreased to 5.7 g/dL, which prompted a blood transfusion, and Gastroenterology was consulted. She underwent an upper endoscopy and colonoscopy, which yielded a small hiatal hernia as well as a few scattered diverticula of mild severity without evidence of bleeding.

**Figure 1:**
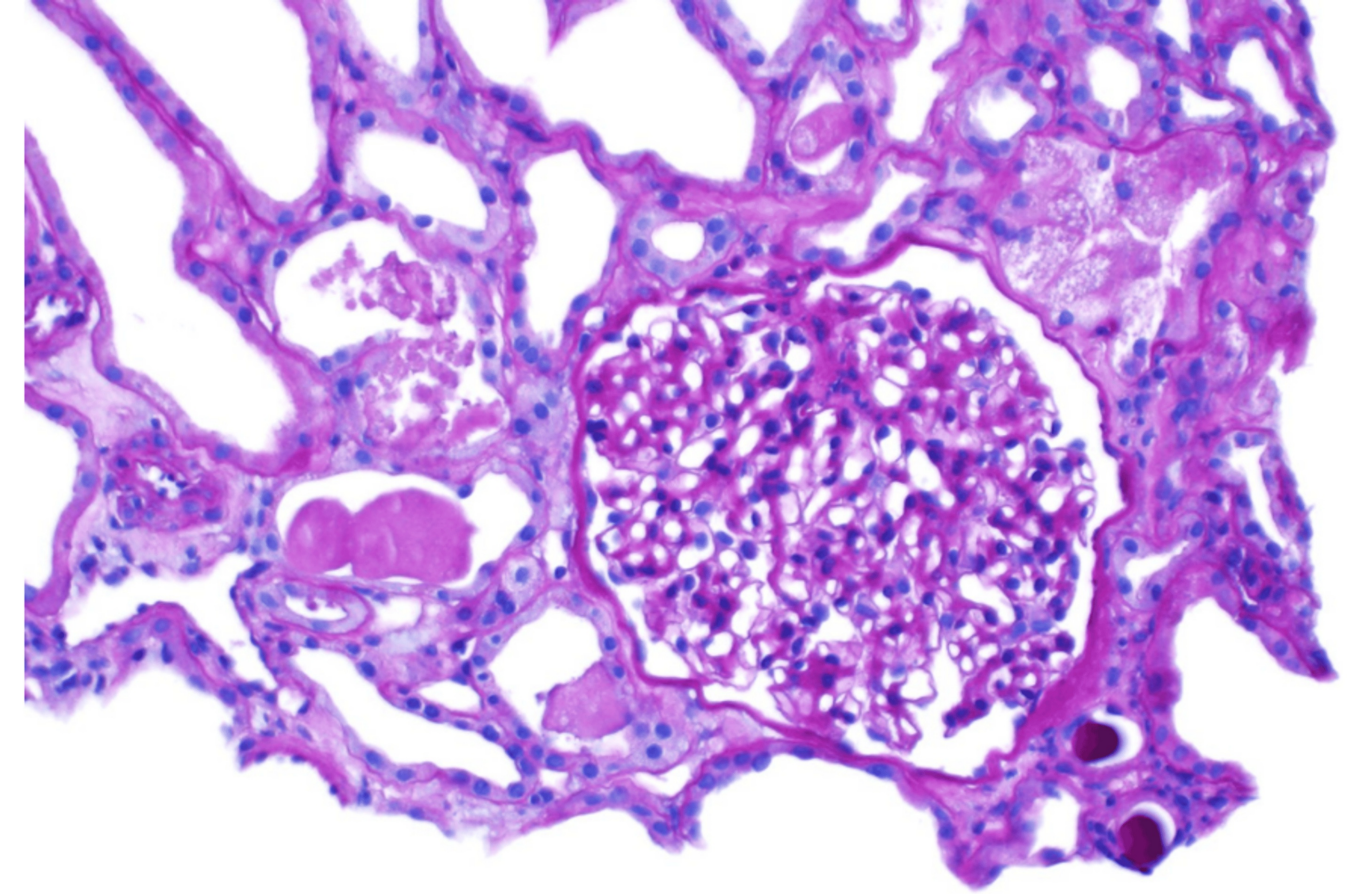
This is a light microscopy image stained with periodic acid-Schiff. The glomerulus is mildly enlarged with mild mesangial matrix expansion without hypercellularity or nodule formation. The capillary loops are without holes, spikes, or double contours. There is no endocapillary hypercellularity, fibrinoid necrosis, or crescents.

**Figure 2:**
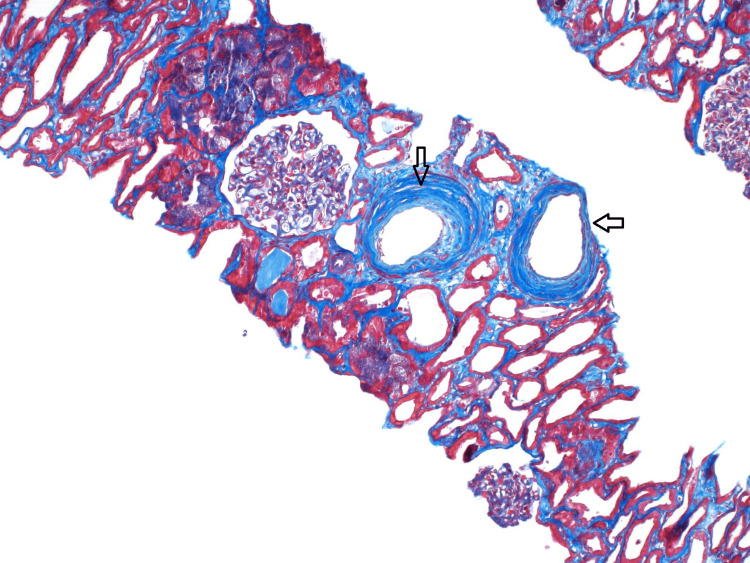
This is a light microscopy image stained with Masson trichrome showing arteries with severe intimal fibrosis (arrows). The dark blue areas between the tubules are in keeping with interstitial fibrosis.

**Figure 3:**
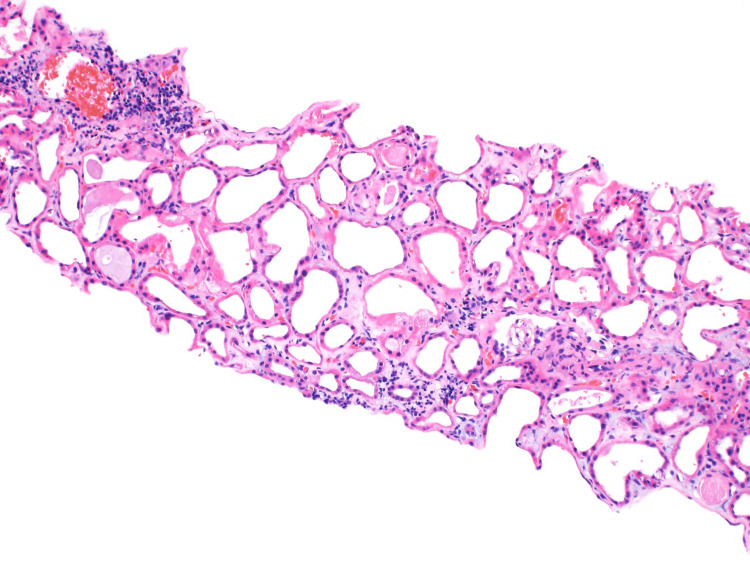
This is a light microscopy image stained with hematoxylin and eosin. There is no significant interstitial inflammation. The tubules are dilated and lined by a markedly attenuated, vacuolated, and reactive epithelium. This is in keeping with acute tubular injury.

**Figure 4:**
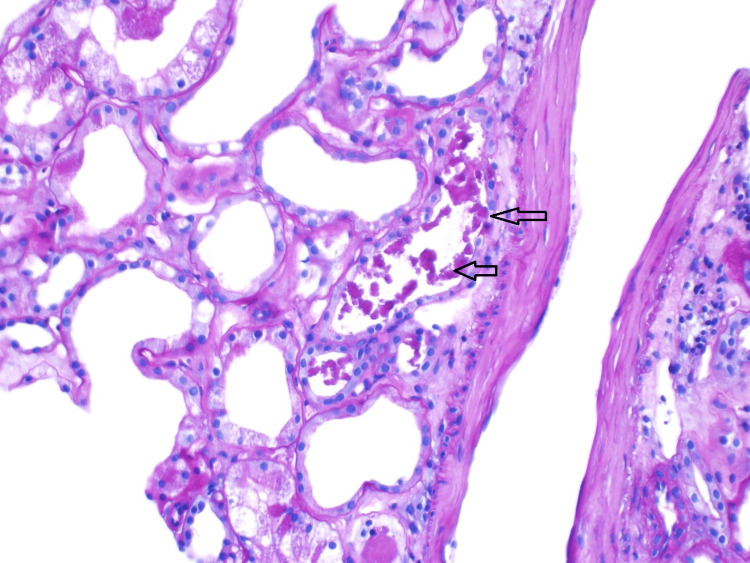
This is a light microscopy image stained with periodic acid-Schiff. The tubular lumen shows frequent globular pigmented casts, shown with the arrows.

**Figure 5:**
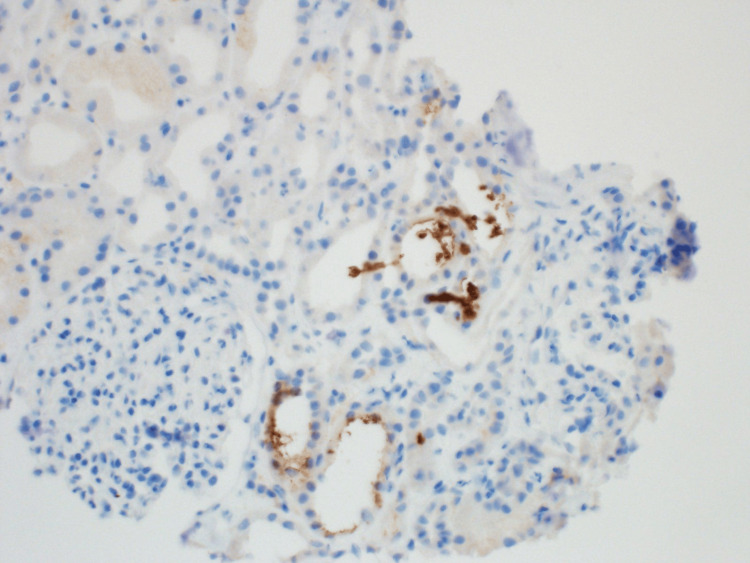
These casts stained positive for myoglobin but were negative for hemoglobin by immunoperoxidase.

Renal biopsy (Figures 1-5) yielded acute tubular injury (ATI) with positive staining for myoglobin casts and negative staining for hemoglobin. There was also diabetic glomerulopathy Class 2A. Immunofluorescence was negative for IgG/M/A, C3, and C1q. Kappa and lambda stained equally throughout. Electron microscopy showed moderately effaced foot processes, and tubular basement membranes were without immune complexes. Congo red staining was negative for amyloid.

The authors acknowledge that on reviewing the delayed ANCA and anti-GBM results and also on receiving the results of the kidney biopsy, the steroid treatment was not indicated. The patient, by day 3 of hospital admission, after commencing dialysis and receiving steroids, had improvement in both urine output with clear color. The patient required three sessions of hemodialysis (HD), after which she began to recover renal function and was discharged to follow-up at an outpatient dialysis center. Her weakness in presentation had also resolved as she was ambulating independently. Three weeks after hospital discharge and during her follow-up, she was able to remain off dialysis, and her creatinine had improved to 1.66 mg/dL.

## Discussion

The clinical presentation of rhabdomyolysis usually incorporates myalgia, weakness, tea or dark-colored urine, and oligo/anuria. This is an atypical case of biopsy-proven myoglobin-induced ATI in the setting of normal CPK and urine myoglobin at the time of biopsy. CPK levels above five times the upper limit of normal are what usually confirm rhabdomyolysis, approximately 1,000 IU/L [1], and the risk for AKI tends to be elevated with levels > 15,000 IU/L [9], although Zahler et al. showed the risk was increased with moderate elevations (>1,000-5,000 IU/L) [10].

Rhabdomyolysis causes the release of myoglobin into the circulation, which is known to be toxic to renal tubules. Myoglobin levels above 1.5 mg/dL surpass the renal threshold, after which it appears in the urine [11]. Myoglobin, especially in the setting of volume depletion and aciduria, exerts its cytotoxic effects where it precipitates with Tamm-Horsfall protein, forming intratubular casts. This myoglobin cast results in tubular obstruction, where blood flow and glomerular filtration rate are decreased. Myoglobin also exerts local cytotoxic effects via the production of free oxygen radicals, which lead to acute tubular necrosis (ATN) [12].

The case presented had myalgia, body weakness, and brown-colored urine, which suggested rhabdomyolysis, but the negative CPK and absence of urine myoglobin argued against this. Even though there were mild diabetic changes seen on biopsy and 10% of interstitial fibrosis and tubular atrophy, there was undoubtedly myoglobin-induced ATI, as the immunohistochemical stain for myoglobin is quite specific. There were no other identifiable causes for her ATI, and the biopsy suggested a recent history of rhabdomyolysis. Her blood urea nitrogen (BUN) was not significantly elevated on presentation at 40 mg/dL. A low BUN-to-creatinine ratio has been seen in patients with rhabdomyolysis [13], and in this patient, it also made prerenal azotemia unlikely. The patient’s long-standing history of anemia and history of cholecystectomy suggest that there may be an underlying hemoglobinopathy. The presence of pigment casts on the renal biopsy meant that the pigment nephropathy could have been from either rhabdomyolysis or hemoglobin. It was therefore important that staining for hemoglobin was done and was negative, solidifying the diagnosis of myoglobin-induced ATI.

The cause of rhabdomyolysis in this patient remains uncertain. What was interesting in this patient was that she had hypokalemia despite her AKI. Hypokalemia has been shown to be a potential trigger for rhabdomyolysis through the process of relative ischemia that leads to muscle cramps and subsequent necrosis [14]. CPK, however, would be expected to be or remain elevated in hypokalemia-induced rhabdomyolysis, and given that CPK was normal on presentation, this made this unlikely. The working hypothesis is that rhabdomyolysis complicated with AKI was in the setting of chronic use of rosuvastatin and the influenza vaccine, which potentially was the trigger. Rosuvastatin is generally considered to be safe. Clinical trials showed that three in 10,000 acquired severe myopathy [15], but in later years, higher rates were seen with those greater than 75 years of age and if there is concomitant use of drugs that interact with rosuvastatin, such as fibrates [16]. This patient first started experiencing symptoms after her flu vaccine. The first reported case of vaccine-induced rhabdomyolysis was by Raman et al. [7], where the patient was a renal transplant recipient on statin and cyclosporine who developed rhabdomyolysis and acute renal failure after the influenza vaccine. Since then, there have been several other reports of acute renal failure triggered by influenza vaccination, where all these patients were also on statin therapy [17-21]. These cases, except for Novati et al. [20], had rhabdomyolysis as the etiology of AKI. This case is no different, but the mechanism by which the influenza vaccine triggers rhabdomyolysis in the setting of chronic statin use is yet to be determined.

There are some limitations in this case report that the authors must acknowledge. Firstly, some investigations could not produce immediate results, which made the initial diagnosis uncertain. ANCA, anti-GBM, and the kidney biopsy results became available a few days into the hospital admission. As a consequence, a second limitation must be acknowledged where the empirical use of steroids was not indicated. This highlights the importance of timely investigations and heightens the consideration of myoglobin-induced tubular injury in very atypical presentations of AKI.

## Conclusions

This is a case in which late presentation of rhabdomyolysis complicated by AKI could have been easily missed, as CPK and urine myoglobin levels were normal, and the diagnosis was made only by renal biopsy and staining for myoglobin. Clinicians and nephrologists should always keep in mind the differential of myoglobin-induced ATI even in the setting of normal CPK. Although influenza vaccine-induced rhabdomyolysis is exceedingly rare, clinicians need to be aware of the risk it may impose on chronic statin users, as it has become a standard of care for high-risk patients to receive this vaccine.
